# Cysteine Proteases and Mitochondrial Instability: A Possible Vicious Cycle in MS Myelin?

**DOI:** 10.3389/fncel.2020.612383

**Published:** 2020-12-01

**Authors:** Anthony Poerwoatmodjo, Geert J. Schenk, Jeroen J. G. Geurts, Antonio Luchicchi

**Affiliations:** Division Clinical Neurosciences, Department of Anatomy and Neurosciences, Amsterdam Neuroscience, Amsterdam Universitair Medische Centra (UMC), Location Vrije Universiteit (VU) Medical Center, MS Center Amsterdam, Amsterdam, Netherlands

**Keywords:** multiple sclerosis, axo-myelinic synapse, cysteine proteases, mitochondria, myelin and axon breakdown, myelin associated glycoprotein

## Introduction

Multiple sclerosis (MS) is a chronic neuroinflammatory disease of the central nervous system (CNS) of which the main pathological feature is the loss of white matter myelin (demyelination) (Ringold et al., 2005).

Although a solid cause to explain MS origin is still debated, a classic etiological view (named *outside-in* hypothesis of MS) regards this disorder as primarily mediated by a faulty CD4^+^ T-lymphocytic attack against the myelin (Lassmann and Ransohoff, 2004; Chitnis, 2007).

Despite its prevalence, this hypothesis has been recently questioned by an opposing view (*inside-out* hypothesis of MS) which posits that subtle primary cytodegenerative processes might happen in the CNS itself, leading to myelin disintegration and a secondary immune system response (Stys et al., 2012). For instance, studies have shown that MS pathological correlates, like widening of myelin lamellae and oligodendrocyte apoptosis, are often present in regions separated form inflammation foci (Barnett and Prineas, 2004; Henderson et al., 2009), and that loss of inner myelin sheath-expressed proteins (like the myelin associated glycoprotein, MAG) temporarily precedes that of outer-sheath expressed molecules in newly forming lesions (Aboul-Enein et al., 2003). Furthermore, clinical observations have recurrently reported that immunosuppressant agents, elective to treat relapsing-remitting forms of MS, are largely ineffective in halting less inflammatory progressive forms of the disease (Stys et al., 2012).

**Figure 1:**
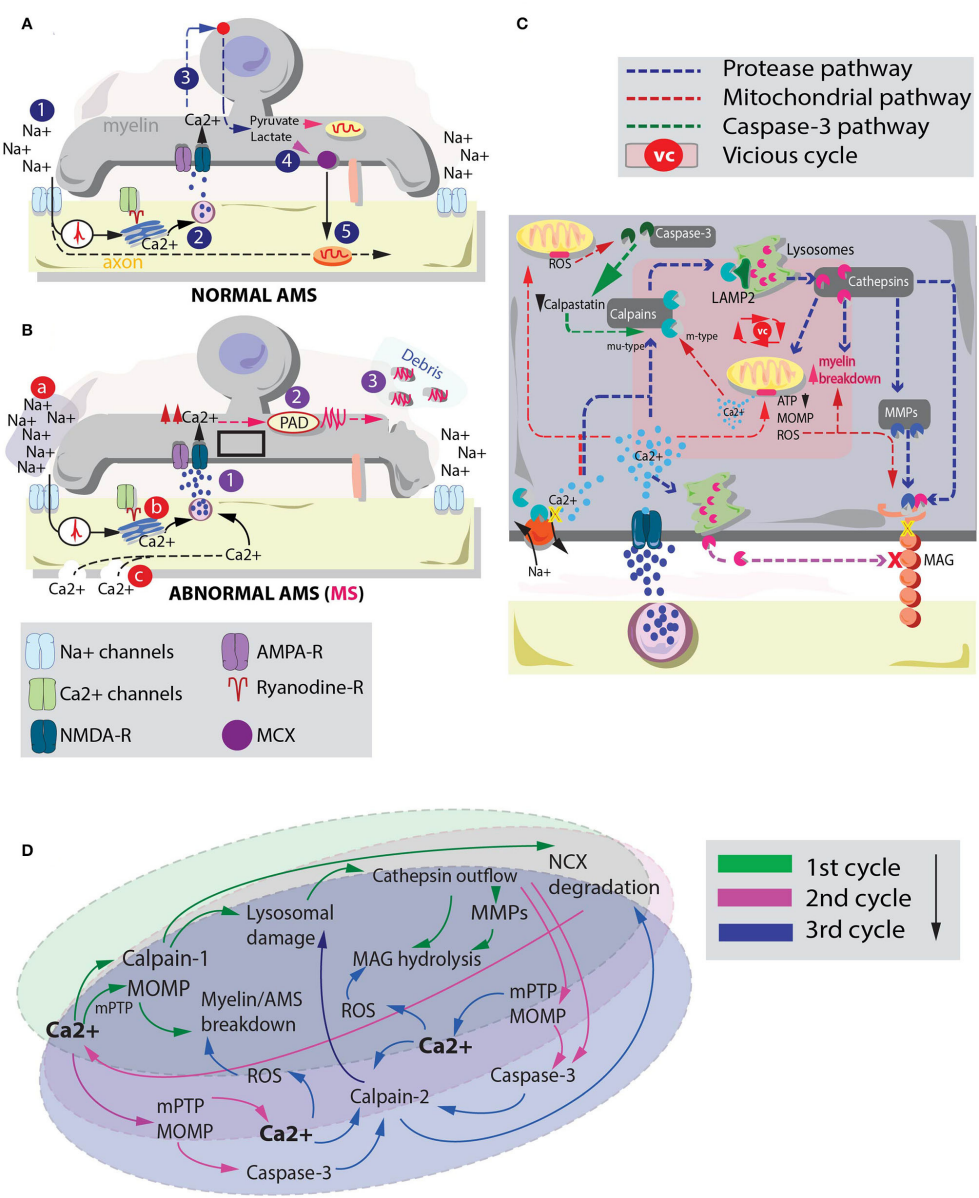
Interplay between cysteine protease activation and mitochondrial deteriorating processes in MS myelin degeneration. **(A)** graphical depiction of the AMS in physiological conditions (Micu et al., 2018). The action potential (1) is sensed by Ca^2+^ channels which via a ryanodine receptor-dependent mechanism mobilize the axoplasmic Ca^2+^ reserves. Ca^2+^ triggers vesicle fusion and glutamate release (2). Glutamate activates NMDA-Rs promoting myelinic Ca^2+^ influx and glycolysis processes. This sequence of events leads to pyruvate and lactate production (3). Lactate is ultimately transported to the axon to metabolically assist axonal mitochondria. **(B)** In pathological conditions, excessive Ca^2+^ myelinic influx is promoted via altered stimulation of NMDA receptors (Micu et al., 2018). Possible players involved in such effect are: (a) an altered Na^+^ homeostasis, where augmented extracellular Na^+^ may lead to intra-axonal aberrant processes that promote Ca^2+^ influx (Craner et al., 2004); (b) altered Ca^2+^ mobilization inside the axoplasmic reticulum (Micu et al., 2018) and (c) the presence of subtle axonal nanoruptures which make axonal cylinder permeable to Ca^2+^ (Witte et al., 2019). Irrespective of the mechanism by which altered Ca^2+^ entrance in the myelin is triggered, such phenomenon (1) may lead to a consequent activation of PADs (2). PADs citrullinate myelin proteins (e.g., MBP). Myelin degenerative processes together with citrullination of MBP promote biochemically altered (highly immunogenic) debris formation (3). **(C)** Summary of the principal pathways by which cysteine proteases (blue pathway) and dysregulated mitochondria (red pathway) can affect myelin and MAG stability. These processes and their interplay may be responsible of myelin and axonal breakdown in MS. Pink box inside myelin depicts the players of a possible vicious cycle between cysteine protease activation and mitochondrial instability in MS-related myelin breakdown. **(D)** Schematic representation of a possible vicious cycle activated by enhanced myelinic Ca^2+^ entrance. Primary Ca^2+^ entrance instigates a parallel activation of mu-type-calpains (calpain-1) and mitochondria degenerative processes. Secondary Ca^2+^ accumulation due to Na^+^/Ca^2+^ exchanger (NCX) degradation and Ca^2+^ efflux from mitochondria might take part in a tertiary activation of m-type calpains (calpain-2) which can exacerbate lysosomal and mitochondria instability leading to heightened myelin damage.

Although a solid sequence of events is still elusive, one putative condition to explain this primary myelin degeneration in MS may be the subtle imbalance at the level of the axon-myelinic synapse (AMS, Figure 1A; Micu et al., 2018). The AMS is a recently proposed form of glutamate-mediated communication between axon and myelin which regulates axonal myelination and action potential propagation via n-methyl-d-aspartate (NMDA) receptor-dependent myelin Ca^2+^ supply (Figure 1A, 1–3; Micu et al., 2018). Briefly, controlled myelinic Ca^2+^ influx takes part in glycolysis processes which lead to pyruvate and lactate production (Figure 1A, 4). Lactate is then back-transported to the axon where it boosts energy production during electric activity (Figure 1A, 5).

Consequently, alterations at the level of the AMS, triggered by aberrant stimuli like dysfunctional Ca^2+^ mobilization from the axoplasmic reticulum (Micu et al., 2018), Na^+^ homeostasis alterations (Inglese et al., 2010) and/or the pathological presence of axonal nanoruptures (Witte et al., 2019) (Figure 1B, a–c) may instigate an augmented myelinic Ca^2+^ entrance. As elegantly shown in studies employing cuprizone mouse models of MS, this condition is responsible of many aberrant biochemical processes relevant for myelin stability including the citrullination of the myelin basic protein (MBP) (Caprariello et al., 2018). Citrullination is a posttranslational modification process instigated by the activation of the enzyme protein arginine deiminase (PAD) that promotes the formation of highly immunogenic myelin (Figure 1B, 1–3).

Several parallel aberrant processes may lead citrullinated myelin to degrade. Among them, the activation of cysteine proteases like the Ca^2+^-dependent non-lysosomal protease calpain and the lysosomal Ca^2+^-independent cathepsin might very well-recapitulate MS myelin degenerative processes.

Calpain and cathepsin play a role in apoptotic processes (Leist and Jaattela, 2001; Kroemer and Jaattela, 2005), Na^+^/Ca^2+^ exchange pump/actin cytoskeleton degradation (McConkey, 1998; Zong and Thompson, 2006), and myelin retraction from the node of Ranvier (Baraban et al., 2018). Moreover, studies on ischemia and sporadic Creutzfeldt-Jakob disease have shown that, under specific circumstances (e.g., aberrant cellular Ca^2+^ influx), these two proteases engage into a sequential activation named “*calpain-cathepsin axis*” where calpain promotes a cleavage of lysosomal associated membrane 2 (LAMP2). As a result, a programmed cell death through lysosomal cathepsin leakage is activated (Miyazawa et al., 1998; Zong and Thompson, 2006; Villalpando Rodriguez and Torriglia, 2013; Llorens et al., 2017).

This axial activation, combined with the hypothesized role of cathepsin in degrading the MAG (Stebbins et al., 1997), an inner-sheath adhesion molecule important for axon-myelinic stability (Trapp and Quarles, 1982; Pronker et al., 2016), may explain the primary degenerative processes involved in MS-related AMS instability and myelin disintegration.

Despite this, whether an aberrant cysteine protease activation is relevant and alone sufficient to explain demyelination in MS is still not understood.

In this perspective article we evaluate the possibility that a disruptive activation of the calpain-cathepsin axis in MS myelin can be reinforced by the activation of parallel Ca^2+^-dependent aberrant events to induce primary myelin degeneration.

One that seems very relevant for MS is the cascade generated by Ca^2+^-dependent mitochondrial dysfunctions (Nicholls, 2009). Mitochondria instability is not a new concept in MS pathology (Witte et al., 2014). In particular, Ca^2+^-mediated aberrant events in the mitochondria, such as the opening of the mitochondria permeability transition pore (mPTP), trigger the release of intramembrane proteins (like cytochrome c) which, in turn, activate an apoptotic process named mitochondrial outer-membrane permeabilization (MOMP) (Ichas and Mazat, 1998).

Interestingly, cysteine protease activation and mitochondria pathological events share a number of common pathways. The activation of these pathways might instigate a “*vicious cycle*,” strongly contributing to explain MS-related myelin degeneration. Here we propose that in MS, following excessive myelinic Ca^2+^ influx, calpain-cathepsin axis activation and MOMP play a synergic role in AMS destabilization and MAG degradation.

## A LOOK AT THE COMPONENTS OF THE CYCLE: CYSTEINE PROTEASES, MITOCHONDRIA AND MAG DEGRADATION

Cysteine proteases actively promote protein catabolism inside the cellular compartment (Verma et al., 2016). Among them, calpain co-exists in two main isoforms: *calpain-1* (mu-type) and *calpain-2* (m-type, which requires higher Ca^2+^ concentrations to be activated) (Yamashima, 2004; Potz et al., 2016). Similarly, cathepsins are present in different forms (Turk et al., 2012).

Besides their role in cellular degeneration, some of these proteases seem particularly relevant for MS pathology. For instance, the L form of cathepsin is thought to play a role in MAG hydrolysis, truncating this molecule into a less functional version named dMAG (Stebbins et al., 1997). Notably, a selective MAG loss is spotted in the lesion formation in several neurodegenerative diseases including Kearns-Sayre syndrome (Lax et al., 2012) and MS (Aboul-Enein et al., 2003). Therefore, a dMAG formation operated by a calpain-cathepsin axis activation may hold important consequences for AMS stability, recapitulating the dynamic of AMS destabilization/myelin-axonal degeneration in MS.

Although the biochemical steps that lead to MAG degradation are not entirely understood (Paivalainen et al., 2003), studies have proposed that cathepsin-dependent MAG truncation may happen via different pathways. One implies a direct effect of cysteine proteases on MAG intracellular domains (Stebbins et al., 1997), while another mechanism operates via an indirect Ca^2+^-dependent lysosomal fusion with the plasma membrane. The latter promotes cathepsin secretion through vesicular exocytosis (Rodriguez et al., 1997; Hashimoto et al., 2015). Finally, cathepsins are also shown to activate matrix metalloproteases (MMPs) (Milward et al., 2008). This third pathway may influence MAG stability through an extracellular matrix degradation and increase in myelin motility (Kroemer and Jaattela, 2005; Gu et al., 2015). Altered myelin motility may profoundly affect the ability of axon and myelin to establish a rigidly regulated point-to-point synapse (Micu et al., 2018), hampering the metabolic coupling they share (Beirowski et al., 2014; Micu et al., 2018; Figure 1C).

Mitochondria dysfunctionalities may corroborate the effects of these proteases on MS myelin. For instance, MOMP is shown to induce a cascade of deteriorating events such as those catalyzed by caspase-9 and−3 (Parsons and Green, 2010). Additionally, due to the role of mitochondria in Ca^2+^ storage, instability at the level of these organelles is able to instigate a detrimental extra-mitochondrial Ca^2+^ release (Montero et al., 2001). The latter effect might add up to the effects caused by augmented NMDAR-dependent myelinic Ca^2+^ influx.

On top of these processes, mitochondria respiratory chain defects are thought to cause MAG loss in relatively non-inflammatory environments. For instance, studies have shown that MAG loss is associated with prominent nuclear expression of HIF-1α, a marker for hypoxia-like metabolic tissue injury. HIF-1α can be induced by either mitochondrial increase in intracellular reactive oxygen species (ROS) production (Aboul-Enein et al., 2003) or by impaired mitochondrial respiratory chain functions (Semenza, 2000). Finally, studies on Kearns-Sayre syndrome reported primary MAG loss and consequent demyelination (Lax et al., 2012). The onset of this disorder is thought to be due to primary mitochondrial respiratory chain defects as a result of a single mtDNA deletion. Therefore, it might be possible that also in MS mitochondrial instability instigates MAG loss with consequent induction of AMS instability/myelin degeneration.

## DISCUSSION: THE VICIOUS CYCLE EXPLAINED

Aberrant myelin Ca^2+^ influx in the AMS may be an essential trigger in MS (Micu et al., 2018). Although a clear sequence of events is still ignored, Ca^2+^ dysregulations are a potent source of parallel biochemical processes that may synergistically partake in degenerative conditions. This interplay might generate a vicious cycle where Ca^2+^-mediated events reinforce each other, explaining complex structural and biochemical alterations like those observed in MS brains. Interestingly, in the case of cysteine proteases and mitochondria dysfunctionality, both processes are crucially involved in degeneration/apoptosis (McConkey, 1998; Festjens et al., 2006; Zong and Thompson, 2006). Therefore, a combined activation of these pathways would likely explain the consequent highly immunogenic myelin fragmentation observed e.g., in cuprizone mouse models (Caprariello et al., 2018).

As one of the roles of the AMS is to supply the axonal compartment with lactate for metabolic purposes, a cysteine protease/mitochondrial-dependent myelin breakup can induce a secondary stage of axonal virtual hypoxia leading to axon disintegration (Stys et al., 2012; Micu et al., 2018).

Cysteine proteases and mitochondria might hypothetically interact in several ways to decide the fate of myelin in MS. Following an initial myelinic Ca^2+^ increase a primary co-activation of calpain-1 and mPTP/MOMP might occur (Figures 1C,D). These events may instigate lysosomal cathepsin outflow and primitive myelin breakdown, respectively (Figure 1D, 1st cycle). Elevated levels of cathepsins can either directly or indirectly affect MAG stability (Rodriguez et al., 1997; Stebbins et al., 1997; Milward et al., 2008; Hashimoto et al., 2015). Furthermore, simultaneously altered level of calpains can promote a cleavage of the Na^+^/Ca^2+^ exchange pump (NCX) (McConkey, 1998; Zong and Thompson, 2006). The latter phenomenon facilitates an additional intracellular Ca^2+^ accumulation which together with the ability of cathepsin-L and D to activate caspase-3 (Li et al., 1997; Yamashima, 2000) and MOMP (Kroemer and Jaattela, 2005) might greatly reinforce the cysteine protease/mitochondria vicious cycle (Figure 1D, 2nd cycle). In fact, caspase-3 is thought to be able to cleave calpastatin, an endogenous calpain inhibitor (Porn-Ares et al., 1998; Yamashima, 2004), instigating a protracted permanence of calpains in the myelinic compartment. Cleavage of calpastatin together with mPTP-dependent mitochondria Ca^2+^ release (De Marchi et al., 2014) might promote the activation and accumulation of other forms of degenerative calpains, like calpain-2, which require Ca^2+^ in the mM range to be activated (Baudry and Bi, 2016). Finally, parallel mitochondria disruptions caused by Ca^2+^ overload can induce ROS enhancement (Festjens et al., 2006) strongly adding up to the ongoing AMS instability, myelin breakdown and MAG degradation (Figure 1D, 3rd cycle and beyond).

## CONCLUDING REMARKS

In this opinion article we proposed a scenario where a primary altered AMS stability might explain myelin breakdown and axonal degeneration in MS via Ca^2+^-mediated aberrant events (Micu et al., 2018). Possible instigators of such processes are the cysteine proteases and mitochondria dysfunctionalities. Here we posit that these two players reinforce each other leading to a possible vicious cycle which may hold important consequences for myelin stability. To confirm this hypothesis rigidly controlled experiments should define whether an actual interplay between the activation of the calpain-cathepsin axis and MOMP exists and, in this case, whether one pathway temporarily precedes the other one.

## Author Contributions

AP, GS, and AL: conceived the study. AP: performed the literature research. GS and JG: provided critical revision to the manuscript. AP and AL: wrote the manuscript with input from all the authors.

## Conflict of Interest

The authors declare that the research was conducted in the absence of any commercial or financial relationships that could be construed as a potential conflict of interest.
